# ^18^FDG-PET/CT for prognostic stratification of patients with multiple myeloma relapse after stem cell transplantation

**DOI:** 10.18632/oncotarget.2290

**Published:** 2014-07-31

**Authors:** Constantin Lapa, Katharina Lückerath, Uwe Malzahn, Samuel Samnick, Herrmann Einsele, Andreas K. Buck, Ken Herrmann, Stefan Knop

**Affiliations:** ^1^ Department of Nuclear Medicine, Medical Center, University of Würzburg, Würzburg; ^2^ Institute of Clinical Epidemiology and Biometry, University of Würzburg, Würzburg; ^3^ Department of Hematology and Oncology, Medical Center, University of Würzburg, Würzburg; ^4^ Clinical Trial Center Würzburg, University Hospital Würzburg, Würzburg

**Keywords:** Multiple myeloma, molecular imaging, FDG-PET/CT

## Abstract

The aim of this study was to investigate the prognostic value of 18F-fluorodeoxyglucose positron emission tomography–computed tomography (18F-FDG-PET/CT) in 37 patients with a history of multiple myeloma (MM) and suspected or confirmed recurrence after stem cell transplantation (SCT). All patients had been heavily pretreated. Time to progression (TTP) and overall survival (OS) were correlated to a number of different PET-derived as well as clinical parameters. Impact on patient management was assessed.

Absence of FDG-avid MM foci was a positive prognostic factor for both TTP and OS (p<0.01). Presence of >10 focal lesions correlated with both TTP (p<0.01) and OS (p<0.05). Interestingly, presence of >10 lesions in the appendicular skeleton proved to have the strongest association with disease progression. Intensity of glucose uptake and presence of extramedullary disease were associated with shorter TTP (p=0.037 and p=0.049, respectively). Manifestations in soft tissue structures turned out to be a strong negative predictor for both, TTP and OS (p<0.01, respectively). PET resulted in a change of management in 30% of patients.

Our data underline the prognostic value of 18F-FDG-PET/CT in MM patients also in the setting of post-SCT relapse. PET/CT has a significant impact on patient management.

## INTRODUCTION

Multiple myeloma (MM) accounts for approximately 1% of all cancers and around 10% of hematological malignancies [1, 2]. Although overall survival has improved over the last decade, MM essentially remains incurable. Besides conventional chemotherapeutic agents, an increasing number of ´novel` drugs have been evaluated, including the proteasome inhibitor bortezomib, the anti-angiogenic agent thalidomide and its immunomodulatory derivatives lenalidomide and pomalidomide [3]. A significant survival benefit has been reported for autologous stem cell transplantation (SCT) [4]. Allogeneic stem cell transplantation exploits immune surveillance of myeloma plasma cells by grafting stem cells derived from a HLA-identical sibling or unrelated donor; however, its application is limited by severe complications as well as graft-versus-host disease that may arise after conditioning and SCT.

Several studies have demonstrated the utility of molecular imaging using positron emission tomography (PET) and the radiolabeled glucose analog ^18^F-2`-deoxy-2`-fluorodeoxyglucose (^18^F-FDG) for diagnosis, staging and estimation of prognosis, leading to implementation into the revised Salmon/Durie staging system (Salmon/Durie PLUS) [5-11]. However, most studies assessing the prognostic implication of PET have focused on early therapeutic settings prior to SCT [12-14]. In a study enrolling 77 patients with previous autologous SCT, *Nanni* and co-workers found a negative PET scan to be predictive for a longer disease-free survival. On the other hand, patients with persisting myeloma activity, as indicated by PET, had a significantly shorter time to relapse [15]. The value of molecular imaging in the setting of relapse after SCT has not been examined yet. Recently, the International Myeloma Working Group emphasized the need for further studies before PET could be recommended as a standard tool in both diagnosis and follow-up of MM patients [16].

The aim of this study was to investigate the prognostic role of ^18^F-FDG-PET in patients with multiple myeloma relapse after autologous and/or allogeneic stem cell transplantation. Impact of ^18^F-FDG-PET on patient management was also assessed.

## RESULTS

### Imaging characteristics

**Figure 1:**
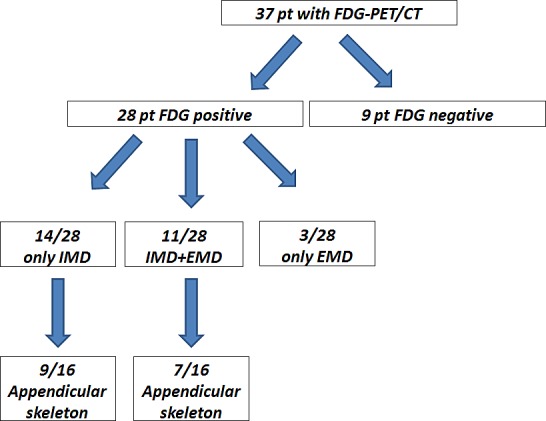
Lesion detection by PET/CT in posttransplant myeloma patients Out of 37 patients undergoing ^18^F-FDG-PET/CT, 28 (76%) had a positive scan. 14 out of those 28 (50%) presented with disease confined to the intramedullary compartment (IMD = intramedullary disease), 11/28 patients (39%) suffered from both intra- and extramedullary disease (IMD + EMD) and the remaining 3/28 patients (11%) had exclusively extramedullary MM manifestations. 16 patients (57%) also showed involvement of the appendicular skeleton, with 7/16 (44%) also presenting with EMD. In 9/37 patients (24%) with serological relapse, no metabolically active MM lesions could be detected.

**Figure 2:**
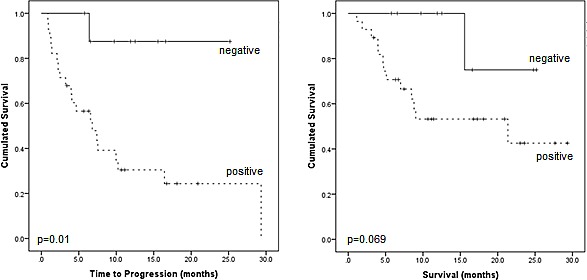
Prognostic impact of PET-positivity on time to progression and overall survival Kaplan-Meier analysis of patient outcome according to presence of PET-positive lesions.

Out of 37 patients with serologically proven relapse from multiple myeloma, 28 (76%) had a positive PET scan. Fourteen out of those (50%) presented with disease confined to the bone marrow compartment (intramedullary lesions), 11 patients (39%) suffered from both intra- and extramedullary disease and the remaining 3 patients (11%) had exclusively extramedullary MM manifestations, resulting in 50% (14/28) of PET-positive patients with extramedullary manifestations. Regarding EMD, lymph node involvement was most commonly seen (11/14, 79%), followed by manifestations of soft tissue (6/14, 43%), liver (4/14, 29%), spleen (2/14, 14%) and lungs (2/14, 14%). Regarding intramedullary MM, 16/28 PET-positive patients (57%) also showed involvement of the appendicular skeleton, with 7/16 (44%) also presenting with EMD. In 9 patients (24%) with serological relapse, no metabolically active MM lesions could be detected (Figure 1). PET negativity turned out to be a positive prognostic factor: In the group of patients with a negative ^18^F-FDG scan, median TTP was not reached (*vs*. 6.8±2.0 m in PET-positive patients; p=0.01) and only one of those subjects deceased not MM-related (endocarditis) during follow-up (OS not reached; *vs*. 21.4±10.4 m for PET-positive patients; p=0.069; Figure 2).

### Number and location of intramedullary lesions

**Figure 3:**
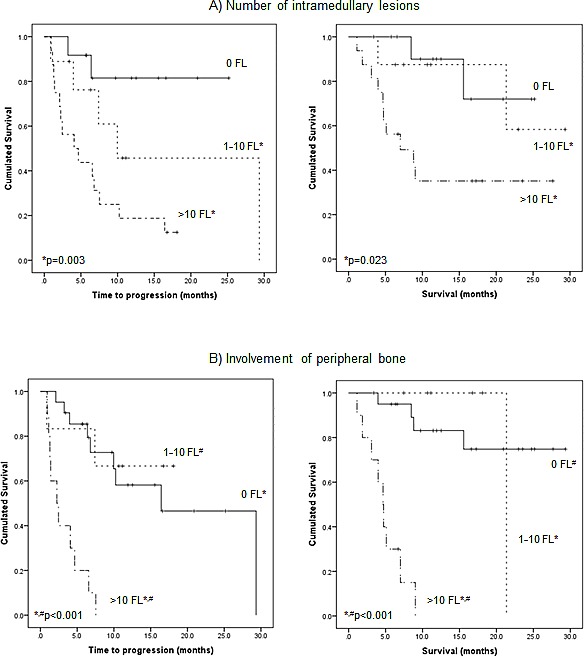
Prognostic impact of number and location of intramedullary lesions on time to progression and overall survival A) Kaplan-Meier analysis of outcome according to the number of PET-positive intramedullary lesions. B) Kaplan-Meier analysis of outcome according to the number of PET-positive focal lesions (FL) in the appendicular skeleton.

Of the 25/37 (68%) patients who presented with intramedullary focal PET-positive lesions, 9/25 subjects (36%) had 1-10 PET-positive lesions, the remainder (16/25; 64%) showed >10 focal lesions. The number of lesions proved to be a significant prognostic factor. In patients with 1-10 lesions, a significantly longer TTP as well as OS was observed, as compared to those with more than 10 intramedullary lesions. Median TTP was 10.0±6.8 m and median survival was not reached in patients with ≤ 10 lesions, whereas TTP was 4.1±2.2 m and OS 7.0±3.3 m (TTP p=0.003; OS p=0.023) in patients with >10 intramedullary lesions (Figure 3A).

In the subgroup of patients with involvement of the appendicular skeleton, 6/16 patients (37%) had 1-10 lesions and 10/16 (63%) had >10 lesions. Here, a high number of lesions was associated with significantly shorter TTP (2.2±0.8 months; p<0.001) and OS (4.7±0.6 months; p<0.001). In the cohort with less than 10 lesions, overall median TTP was significantly longer 10.0±2.2 m (p<0.001) and OS was not reached (p<0.001) (Figure 3B).

### Number and location of extramedullary lesions

**Figure 4:**
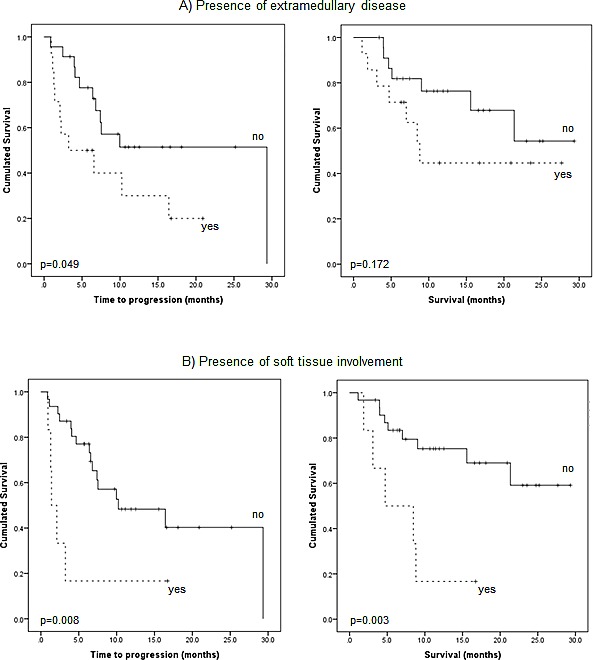
Prognostic impact of extramedullary disease A) Kaplan-Meier analysis of outcome according to presence of extramedullary disease itself. B) Sub-analysis regarding outcome according to presence of soft tissue involvement.

**Figure 5:**
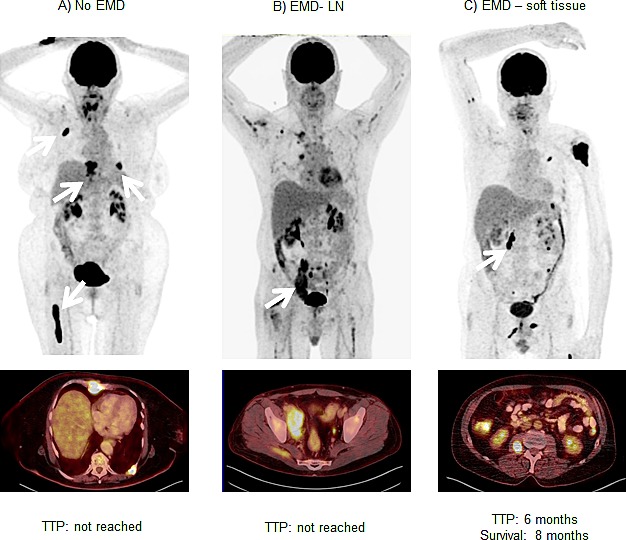
Negative prognostic value of extramedullary soft tissue lesions Display of three patients without (A) and with (B; C) extramedullary myeloma manifestations. Whereas patient A had exclusively intramedullary lesions, patients B and C presented with intramedullary and extramedullary disease. Patient B had mainly iliac lymph node involvement (arrow), patient C suffered from muscle manifestations (arrow). All patients received chemotherapy with novel agents after ^18^F-FDG-PET/CT; follow-up was 8 months in all. Whereas patients A and B have not progressed yet, patient C died 8 months after the PET scan. EMD = extramedullary disease, LN = lymph node, TTP = time to progression

In 14/37 (38%) patients with extramedullary disease, TTP and OS was shorter than in the patients with no EMD (median TTP 3.2±3.4 m *vs*. 29.3±0.0 m, p=0.049; median OS 8.8±1.5 m *vs*. not reached, p=0.172; Figure 4A). However, sub-analysis of disease allocation revealed that presence of myeloma lesions in soft tissue structures (6/37, 16%) was associated with significantly shorter median TTP and median OS (Figure 4B). Subjects with soft tissue involvement turned out to have a median TTP of 1.4±0.5 months (*vs*. 10.2±5.2 m in all patients without soft tissue involvement; p=0.008) and a median OS of 4.7±3.3 months (*vs*. OS not reached; p=0.003). In comparison to patients with EMD and involvement of other organs, absence of soft tissue lesions was associated with an improved median TTP of 10.2±4.0 months (p=0.021) and median OS was not reached (p=0.013). Figure 5 shows an example of soft tissue involvement and limited survival of 8 months despite multidrug therapy including novel compounds. Interestingly, soft tissue involvement was associated with extensive intramedullary disease (>10 intramedullary lesions) in 5/6 (83%) patients. The remaining patient without intramedullary MM suffered from co-existing LN involvement, which occurred in the majority of EMD cases (11/14, 79%). On its own, presence of nodal involvement was associated with a slightly longer median TTP (6.6±3.2 m) and median OS 8.8±0.0 m as compared to EMD without LN involvement (median TTP 2.2±0.76 m, p=0.097; median OS 7.0±1.9 m, p=0.379).

### Activity of lesions

In the 28 patients with a positive PET scan, hottest lesion activity, as assessed by SUV_max_ in intra- and extramedullary lesions, was correlated to TTP or OS. Using ROC analysis, cut-off values designating inferior outcome were derived. A value of SUV_max_>18.57 was predictive for shorter TTP (median 7.0±3.2 m *vs*. not reached; p=0.037). OS could not significantly be related to lesion activity. However, there was a trend to inferior survival when lesion activity was >10.36 (p=0.104).

### Predictive value of cytogenetics

In the 23 patients screened for cytogenetic abnormalities, high risk signatures occurred in 9/23 (39%). Out of these, 7/9 (78%) had a positive PET scan, 6/7 (86%) presented with >10 and 1/7 (14%) with ≤10 focal lesions. The remaining patients (2/9, 22%) were PET negative as compared to 7/14 (50%) subjects with standard risk profiles. EMD could be detected in 4/9 (44%) high-risk patients (*vs*. 3/14 (21%) with standard risk). Overall, unfavorable cytogenetics were also associated with shorter median TTP (6.8±6.8 m, p=0.364) and median OS (9.0±3.03 m, p=0.047). Furthermore, in 6/9 subjects (67%) with high-risk profiles, disease progression was observed (median TTP 6.8±6.8 m; median OS 9.0±3.0 m) whereas only 4/14 (29%) patients with standard risk profiles deceased during follow-up (median TTP 29.3±0.0 m, p=0.108; median OS not reached, p=0.044).

### Predictive value of laboratory characteristics

**Figure 6:**
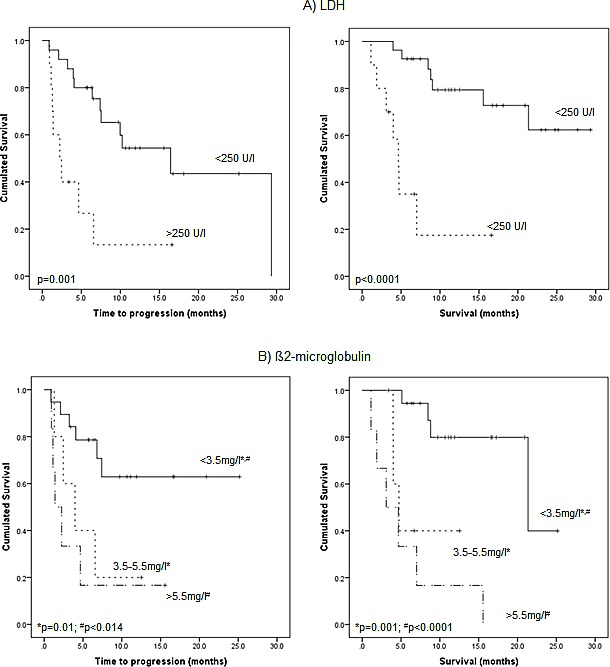

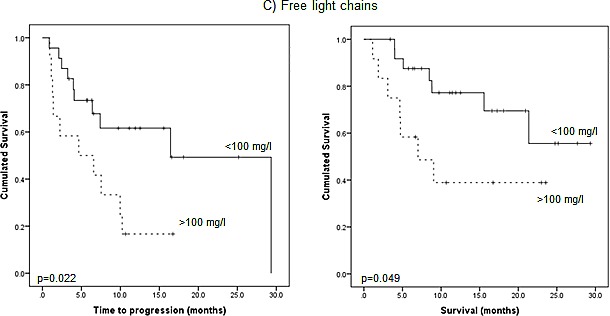
Prognostic impact of biochemical serum parameters: A) lactate dehydrogenase (LDH), B) β2-microglobulin, and C) free light chains Kaplan-Meier analysis of outcome according to presence of elevated levels of LDH (>250 U/l) (A), β2-microglobulin (>3.5 mg/l) (B) and free light chains (kappa or lambda, respectively; >100 mg/l) (C).

Regarding biochemical work-up, 10/37 patients (27%) presented with elevated LDH levels (>250 U/l). Elevated LDH was associated with a positive PET scan (9/10, 90%). Interestingly, 8/9 PET-positive patients (89%) presented with >10 lesions and also had appendicular intramedullary lesions. EMD was present in 60% (6/10). 7/10 patients (70%) with elevated LDH at the time point of PET scanning died (median TTP 2.2±0.84 m, p=0.001; OS 4.7±0.5 m, p<0.0001; Figure 6A).

β2-microglobulin levels were >3.5-5.4 mg/l in 5/30 (16.7%) and >5.5 mg/l in 6/30 (20%) patients. The remaining 19 patients (63%) with available data presented with β2M levels <3.5 mg/l. Elevation of β2M proved to be a negative prognostic factor: In 4/5 (80%) patients with intermediate β2M levels (3.5-5.4 mg/l), the PET scan returned positive findings (3/5 (60%) patients presenting with >10 intramedullary lesions and in 2 patients (40%) with EMD). 4/5 patients (80%) progressed and 3/5 (60%) died during follow-up with a median TTP of 4.0±1.6 m (p=0.01) and OS of 4.7±0.8 m (p<0.001), respectively. In the high-risk group with β2M levels >5.5 mg/l, 5/6 patients (83%) were PET positive and had >10 intramedullary lesions with 4/6 (66.7%) showing EMD. All patients deceased with a median TTP of 1.4±0.7 m (p=0.014) and OS of 3.1±1.7 m (p<0.0001) (Figure 6B).

Free light chains were elevated in every patient. 12/37 (32%) patients had FLC levels >100 mg/l. Severe FLC elevation was strongly associated with a positive PET scan (12/12), with more than 10 focal skeletal lesions in 10/12 (83%) cases. The remaining 2/12 patients (17%) had 1-10 focal lesions. Regarding the appendicular skeleton, >10 lesions were present in 8/12 (67%) cases, 1-10 lesions in 2/12 (17%) cases. EMD was present in 8/12 (67%), as compared to 6/25 (24%) cases with FLC<100.

High-risk cytogenetics were found in 6/12 (50%) patients. Presence of FLC levels >100 mg/l was associated with significantly shorter TTP (median 4.7±3.8 m, p=0.022) and OS (median 7.0±3.3 m, p=0.049; Figure 6C).

### Impact of ^18^F-FDG-PET/CT scans on patient management

In 11 of the 37 cases (30%), PET/CT directly influenced treatment decisions: in 2 patients, PET/CT indicated disease activity which had not been revealed at routine follow-up (diffuse, non-secretory bone marrow relapse and solitary extramedullary manifestation, respectively). In the remaining 9 patients with intramedullary myeloma relapse, previously unknown extramedullary disease was detected *de novo*. In the patient with non-secretory relapse systemic treatment for what previously had been deemed fever of unknown origin was initiated. Treatment intensification (multi-agent chemotherapy and stem cell transplant, respectively) based upon the PET findings was performed in the other 10 patients.

## DISCUSSION

This is the first study to analyze prognostic utility as well as clinical impact of FDG-PET/CT in relapsed multiple myeloma patients after allogeneic and/or autologous SCT. In this unique cohort of 37 subjects, PET negativity was significantly correlated with longer TTP and OS whereas presence of FDG-avid disease, especially a high number of focal lesions (FL) with involvement of the appendicular skeleton and/or extramedullary disease with soft tissue lesions was predictive of unfavorable outcome.

The role of the number of PET-avid FL as an adverse prognostic factor has already been introduced in previously untreated MM patients [13, 14]. Interestingly, in our patient collective-besides the overall involvement of the skeleton- the number of detected lesions (>10) in the appendicular skeleton was particularly correlated with shorter TTP and OS. The impact of the distribution of bone lesions has already been reported for prostate cancer [17-19]. One study investigating 86 prostate cancer patients with bone metastases reported on better prognosis of axial as compared to appendicular metastases [19]. The authors speculated that appendicular involvement portended a worse prognosis due to higher tumor volume. In MM, extension of malignant plasma cells to the extremities might also identify higher tumor burden as well as more aggressive biology.

Presence of EMD has also been established as an independent unfavorable prognostic factor [6, 13, 14]. In our cohort, we could confirm that also in patients after SCT the presence of EMD is associated with shortened TTP (and OS). Interestingly, there was a much stronger correlation with both TTP and OS when extramedullary lesions were present in soft tissue structures. As soft tissue lesions occurred mostly in patients with extensive intra- (and extra-) medullary disease, this might be explained by the presence of very advanced disease.

In parallel to studies enrolling patients with newly diagnosed MM directly prior to or after up-front autologous SCT [12], intensity of FDG uptake was negatively correlated with TTP. However, the threshold of SUV_max_ observed in our series was substantially higher as previously reported (SUV_max_>18.57 *vs.* SUV_max_>4.2). This is partly in line with a recent report that did not find any statistically significant difference in SUV_max_<4.2 after up-front SCT in PET-positive patients who subsequently relapsed and in PET-positive patients who presented with stable disease [15]. This observation might be explained by the late-stage patient collective presenting with more aggressive and thereby highly metabolically active disease. The value of SUV_max_ might reside in treatment assessment but this requires further elucidation.

We additionally investigated the association between established laboratory serum parameters and ^18^FDG-PET. Elevated LDH (>250 U/l), β2M (>3.5 mg/dl) as well as severely elevated FLC levels (>100 mg/l) correlated with a positive PET scan, the number of focal lesions, EMD as well as TTP and OS. Therefore, ^18^FDG-PET/CT might prove especially useful for the treating physician when local therapies-either alone or as an adjunct to systemic treatments- are considered in patients with increasing serum markers: it is able to demonstrate individual entities of disease involvement and thereby stratify prognostic subgroups starting from patients with a negative scan PET scan to subjects with widespread extramedullary disease.

Additionally to its diagnostic value, PET/CT also directly influenced treatment decisions in approximately one third of cases (11/37) leading to treatment initiation (1/11) or intensification (multi-agent chemotherapy and stem cell transplant, respectively (10/11)). By depicting more widespread disease, PET/CT might directly impact treatment approaches in patients who are (still) eligible for therapy and may even benefit from more aggressive therapies.

This study has a number of limitations. First, its retrospective design and low patient numbers limit statistical power. Second, no comparison with conventional imaging modalities such as magnetic resonance imaging (MRI) or CT was performed. This was out of the scope of this study which concentrated on the predictive value of PET. Additionally, MR and CT scans were performed on different scanners using different protocols in some cases, thereby hampering direct comparison. Since not all positive MM lesions, especially extramedullary manifestations, were histologically proven, there might be a false positive rate, e.g. for lymph node involvement. It is not clear if – and to which extent – individual treatment decisions based of PET/CT imaging might have influenced patients' overall prognosis. It could be speculated that switching a given patient to a more “intensive treatment pathway”, based on PET/CT findings, may ultimately result in more favourable outcomes. However, data indicating more intensive systemic therapies being superior to less intensive ones are not unequivocal [20, 21] and are especially not convincing in widespread extramedullary multiple myeloma [22]. In all, treatment-induced bias due to heterogeneous treatments at relapse cannot be excluded in this series and may account for some of the prognostic differences that were found. It is only by a prospective trial that one will be able to eliminate efficacy-related influence.

In summary, our data underline the prognostic value of ^18^F-FDG-PET/CT in multiple myeloma in relapsed patients after SCT. PET seems to be reliable in predicting outcome by depicting the extent of active disease and distinguishing different subgroups of patients with distinct myeloma biology. Additionally, implementation of PET into the diagnostic algorithm has an impact on patient management in about 30% of cases and might raise the opportunity for treatment individualization in the future.

## MATERIALS AND METHODS

Due to the retrospective nature of this study, our institutional review board waived the requirement for informed consent. Still, all patients gave written informed consent to receive ^18^F-FDG-PET/CT imaging for the purpose of restaging and assessment of disease activity.

### Patients

Between September 2010 and August 2013, 37 patients (29 males, 8 females, age 39-75 y, mean 60±9 y) with a history of multiple myeloma were enrolled. All patients suffered from long-standing disease (mean duration 4.7±3.2 y) and had been pre-treated with a number of various chemotherapies, including novel agents such as bortezomib, lenalidomide and others. Radiation therapy had been administered to 28/37 patients (76%). 35/37 subjects (95%) had undergone autologous SCT, 15/37 (41%) primary (2/37) or additional (13/37) allogeneic SCT. Detailed patients´ characteristics are given in Table 1.

**Table 1:** Patients` characteristics

No.	Sex	Age	Myeloma type	Cytogenetics	Disease duration	Previous treatment	Previous auto-Tx	Previous allo-Tx
1	m	60	IgA λ	high risk	7.6	CTx, RTx	yes	yes
2	m	59	IgA λ	n/a	7.9	CTx, RTx	yes	yes
3	f	71	IgG κ	other	9.8	CTx, RTx	yes	none
4	m	51	light chain κ	other	3.1	CTx, RTx	yes	none
5	f	63	light chain κ	other	1.5	CTx, RTx	yes	none
6	m	52	IgG κ	high risk	4.8	CTx, RTx	yes	yes
7	m	54	IgG κ	other	6.8	CTx, RTx	none	yes
8	m	50	IgA κ	other	1.5	CTx, RTx	yes	none
9	m	75	IgG λ	n/a	3.1	CTx	yes	none
10	f	51	IgA κ	other	1.4	CTx, RTx	yes	none
11	m	72	IgG κ	n/a	3.1	CTx	yes	none
12	m	39	IgG κ	n/a	4.7	CTx, RTx	yes	none
13	m	44	IgG κ	other	11.1	CTx, RTx	yes	none
14	m	49	IgA κ	high risk	6.6	CTx, RTx	yes	yes
15	m	54	IgA λ	high risk	2.1	CTx	yes	none
16	m	67	light chain κ	n/a	6.5	CTx	yes	yes
17	f	59	IgG κ	high risk	4.8	CTx, RTx	yes	yes
18	m	50	light chain κ	high risk	1.5	CTx, RTx	yes	none
19	m	51	IgG κ	other	2.9	CTx, RTx	yes	none
20	m	64	IgG λ	n/a	7.5	CTx	yes	yes
21	m	55	IgA λ	other	2.5	CTx, RTx	yes	none
22	m	67	IgG λ	n/a	3.6	CTx, RTx	yes	yes
23	m	57	IgG λ	other	9.4	CTx, RTx	yes	yes
24	m	58	IgG κ	other	0.6	CTx, RTx	yes	none
25	f	64	IgG λ	other	4.0	CTx, RTx	yes	yes
26	m	49	IgA λ	high risk	4.7	CTx	yes	yes
27	m	63	IgG λ	n/a	0.4	CTx, RTx	yes	none
28	m	69	IgG k	other	3.2	CTx, RTx	yes	none
29	m	63	IgA λ	high risk	1.0	CTx	yes	none
30	f	72	Ig G λ	n/a	6.8	CTx, RTx	yes	none
31	m	72	IgG k	n/a	14.2	CTx, RTx	yes	none
32	m	53	Ig A λ	n/a	6.0	CTx, RTx	yes	yes
33	m	69	IgG λ	other	3.6	CTx	yes	none
34	f	65	light chain κ	n/a	5.2	CTx, RTx	yes	none
35	m	52	IgG k	n/a	2.8	CTx, RTx	yes	yes
36	f	75	IgG λ	high risk	0.6	CTx	yes	none
37	m	54	IgG λ	n/a	7.8	CTx, RTx	none	yes

High-risk cytogenetics included the presence of del(17p), t(4;14), t(14;16), t(14;20) and chromosome 1 abnormalities, whereas all other karyotypes were classified as standard risk. Disease duration is given in years. CTx = chemotherapy including novel agents, RTx = radiotherapy. Auto-Tx = autologous stem cell transplant, allo-Tx = allogeneic stem cell transplant. N/A = information not available.

At the time point of PET/CT imaging, recurrent disease was proven or highly suspected due to increased serological disease activity. After PET/CT, patients were monitored regardless of initiated therapies. 16/37 (43%) patients received further systemic therapy, 9/37 (24%) underwent allogeneic SCT, 2/37 (5%) underwent radiation therapy and 1/37 (3%) had combined radiochemotherapy. For the subcohort of survivors, median follow-up was 13 months (range 3-29 months) and for patients who deceased during follow-up, median time-to-death was 5 months (range 1-21 months).

Time to progression (TTP) and overall survival (OS) were correlated to a number of different PET-derived (SUV_mean_; SUV_max_) as well as clinical parameters (lactate dehydrogenase [LDH], β2 microglobulin [β2M], free immunoglobulin light chains [FLC], cytogenetic aberrations).

### Imaging

#### Preparation of ^18^F-FDG

^18^F-FDG was synthesized in house with a 16 MeV Cyclotron (GE PETtrace 6; GE Healthcare, Milwaukee, USA) using GE FASTlab methodology according to the manufacturer's instructions. Before use, radiochemicals were analyzed by HPLC for radiochemical identity and purity.

### PET/CT

PET/CT was performed on a PET/CT scanner (Siemens Biograph mCT 64, Siemens, Knoxville, USA) consisting of a lutetium oxyorthosilicate full-ring PET and a 64-slice spiral CT. ^18^F-FDG (316 ± 30 MBq) was injected intravenously. After a period of 60 min, transmission data were acquired using contrast-enhanced spiral CT (dose modulation with a quality reference of 210 mAs, 120 kV, a 512 × 512 matrix, 5 mm slice thickness, increment of 30 mm/s, rotation time of 0.5 s, and pitch index of 0.8) including the base of the skull to the proximal thighs. Consecutively, PET emission data were acquired in 3D-mode with a 200 × 200 matrix with 2 min emission time per bed position. After decay and scatter correction, PET data were reconstructed iteratively with attenuation correction using a dedicated software (HD. PET, Siemens Esoft, Erlangen, Germany). Standard criteria to define lesions as PET/CT positive were applied according to Zamagni *et al.* [12]. Briefly, presence of areas of focally increased tracer uptake within bones (e.g. more intense compared to normal bone marrow (BM) uptake) excluding articular processes, with or without corresponding lesion identified by CT were rated positive for multiple myeloma; alternatively, a maximum standardized uptake value of SUV_max_ ≥ 2.5 within osteolytic lesions exceeding 1 cm in size or SUV_max_ > 1.5 within osteolytic lesions ranging between 0.5 and 1 cm in size were rated positive. Up to 10 lesions were recorded. If subjects presented with more than 10 lesions (FL), they were categorized into the subgroup >10 FL. Lesions in the appendicular skeleton were divided from those in the axial portions. Diffuse BM involvement was considered if the tracer uptake was diffusely increased with a SUV_max_ equal to or greater than the uptake in the spleen. SUV_max_ of the hottest intramedullary lesion was measured. Presence of extramedullary disease (EMD), defined as FDG-avid lesions that, according to corresponding CT sections, was not contiguous to bone or arose in soft tissue, were described by lesion location and the number of lesions was recorded. Paramedullary disease arising from bone was considered as a myeloma lesion but not as EMD. In parallel to medullary disease, SUV_max_ and location of the hottest extramedullary lesion were recorded.

### Laboratory investigations

At the time point of ^18^FDG-PET/CT scanning, a variety of laboratory parameters were evaluated for their prognostic impact. Free immunoglobulin light chains ([FLC]; all patients) were recorded. Levels of β2-microglobulin ([β2M]; available in 30 patients) and lactate dehydrogenase ([LDH]; all patients) as known prognosticators for an adverse prognosis were documented as well. Additionally, interphase molecular cytogenetics based on fluorescence *in situ* hybridization (FISH) were available in 23/37 patients (62%). Presence of del(17p), t(4;14), t(14;16), t(14;20) and chromosome 1 abnormalities were considered as high-risk, whereas all other karyotypes were classified as standard risk.

### Statistical analysis

Statistical analyses were performed using PASW Statistics software (version 22.0; SPSS, Inc. Chicago, IL). Quantitative values were expressed as mean ± standard deviation or median and range as appropriate. Comparisons of related metric measurements were performed using Wilcoxon-signed rank test. The Chi square- or Fisher exact test was conducted for comparison of frequency data between independent subgroups. Survival probabilities were calculated according to the Kaplan-Meier method and the log-rank test was used for statistical comparison of survival curves between independent subgroups. All statistical tests were performed two-sided and a p-value < 0.05 was considered to indicate statistical significance. No correction of p-values was applied to adjust for multiple tests.
